# Forensic comparison analysis of smokeless powders by gel permeation chromatography and likelihood ratio evaluation methods

**DOI:** 10.1093/fsr/owaf005

**Published:** 2024-02-06

**Authors:** Hongling Guo, Ping Wang, Can Hu, Hongcheng Mei, Yajun Li, Jun Zhu

**Affiliations:** Institute of Forensic Science, Ministry of Public Security of China, Beijing, China; Institute of Forensic Science, Ministry of Public Security of China, Beijing, China; Chinese People’s Public Security University, Beijing, China; Institute of Forensic Science, Ministry of Public Security of China, Beijing, China; Institute of Forensic Science, Ministry of Public Security of China, Beijing, China; Institute of Forensic Science, Ministry of Public Security of China, Beijing, China; Institute of Forensic Science, Ministry of Public Security of China, Beijing, China

**Keywords:** forensic sciences, smokeless powders, gel permeation chromatography, likelihood ratio, molecular weights, polydispersity index

## Abstract

In China, the use of smokeless powders for making homemade ammunition and bombs is an incipient criminal practice. One of the key tasks of analyzing smokeless powders in forensic sciences is to make comparisons between them, providing information about their source or establishing a link between two different smokeless powders seized at different locations. The main component of smokeless powders is nitrocellulose (NC) no matter what type of the smokeless powder is. As a kind of polymer, NC may have different molecular weights and polydispersity index (PDI) values, which could help the identification and differentiation of the smokeless powders. In this study, weight-average molecular weights (Mw), number-average molecular weights (Mn), and PDI value of 79 propellants samples from different sources were measured by gel permeation chromatography, and likelihood ratio evaluation method was applied to facilitate interpretation of the data. The possibility of using these methods to make comparisons between smokeless powders was explored instead of depending on analysis of target compounds with trace amounts in them.

## Introduction

Smokeless powders (SLPs) are often used in civilian and military ammunition. In China, they are the most common low explosives used to fabricate improvised explosive devices such as pipe bombs and home-made ammunition due to their relative ease of accessibility. As a consequence, SLPs are commonly encountered in the investigation of many firearm- and explosive-related crimes, making their examination, analysis and profiling very important from a forensic perspective. It is common to make comparisons between the SLPs used in the devices and those available in suspect’s houses or manufacturing places for forensic investigations. These procedures may help investigators establish a link between two different SLPs seized at different locations.

The major classes of compounds in smokeless powders include energetics, stabilizers, plasticizers, flash suppressants, deterrents, opacifiers, and dyes [1]. According to the main chemical substances in the energetic class of compounds, smokeless powders can be classified into three different types: single-base, double-base, and triple-base. A single-base powder contains nitrocellulose (NC), a double-base powder contains nitrocellulose and nitroglycerin, and a triple-base powder contains nitrocellulose, nitroglycerine, and nitroguanidine. NC is the main component no matter what type of the smokeless powder is. It was reported that infrared spectroscopy, chromatography, and mass spectroscopy have been commonly used for the determination of different ingredients in smokeless powders [2–5]. These above-mentioned methods mainly focus on identifying the additives in SLPs and disregard the main component of NC. Due to the low amount and limited variation of additives across manufacturers and batches [6], the chemical characterization of NC itself might significantly increase the options for forensic comparison and attribution.

NC is a structurally complex polymer that is produced by the nitration of plant-based cellulose using concentrated nitric and sulfuric acid [7]. Different from small molecular compounds, which have exact molecular weight values, polymer molecular weight is defined as a distribution rather than a specific number because polymerization occurs in such a way to produce different chain lengths [8], which is an important feature of polymer compounds. The concept of molecular weight of polymer materials is usually characterized by weight-average molecular weight (Mw) or number-average molecular weight (Mn) and its distribution property, the polydispersity index (PDI). Gel permeation chromatography (GPC) is a useful and robust technique for polymer material analysis and has been investigated for many years [7, 9–11]. However, very few papers have reported the analysis of NC by GPC [7, 12]. In this study, we explore the possibility and suitability using Mw, Mn and PDI data of NC and likelihood ratio (LR) calculation method to make further discrimination and data evaluation between SLPs.

## Materials and methods

### Collection and preparation of samples

We use propellant samples with known sources to test the validity of the methods used. Detailed information, ⁓79 collected smokeless powders were presented in Table 1. The powders were taken out from the cartridges by using a cartridge puller. The SLP samples were dissolved in tetrahydrofuran (THF) at room temperature with a concentration ⁓1 mg/mL and left overnight to dissolve.

**Table 1:** Smokeless powder sample information for measurement.

Sample number	Manufacturer	Cartridge category	Production year	Caliber (mm)	Sample number	Manufacturer	Cartridge category	Production year	Caliber (mm)
s1	121	51 pistol	1958	7.62	s41	81	59 rifle	1966	9.00
s2	121	51 pistol	1963	7.62	s42	911	56 rifle	1968	5.60
s3	121	51 pistol	1964	7.62	s43	911	56 rifle	1979	5.60
s4	121	51 pistol	1965	7.62	s44	C	Sporting rifle	Unknown	5.60
s5	121	51 pistol	1970	7.62	s45	CJ	SS109 rifle	2000	5.60
s6	121	51 pistol	1972	7.62	s46	Czech Republic	Pistol	Unknown	7.65
s7	121	51 pistol	1979	7.62	s47	KKJ	Nail	Unknown	6.80 × 18
s8	121	51 pistol	1981	7.62	s48	KKJ	Nail	Unknown	6.30 × 16
s9	121	51 pistol	1987	7.62	s49	KKJ	Nail	Unknown	6.3 0× 16
s10	121	51 pistol	1991	7.62	s50	LY	Parabellum pistol	1994	9.00
s11	121	51 pistol	2018	7.62	s51	NS	Nail	Unknown	6.80 × 11
s12	121	Revolver pistol	2005	9.00	s52	NS	Nail	Unknown	6.80 × 11
s13	121	DAP92 pistol	2019	9.00	s53	NS	Nail	Unknown	6.80 × 18
s14	121	DAP92 pistol	2018	9.00	s54	NS	Nail	Unknown	6.80 × 18
s15	121	64 pistol	2007	7.62	s55	NS	Nail	Unknown	5.60 × 16
s16	121	64 pistol	1990	7.62	s56	NS	Nail	Unknown	5.60 × 16
s17	121	64 pistol	1990	7.62	s57	NS	Nail	Unknown	6.30 × 16
s18	121	64 pistol	1992	7.62	s58	NS	Nail	Unknown	5.60 × 16
s19	121	64 pistol	1995	7.62	s59	NS	Nail	Unknown	6.80 × 11
s20	121	64 pistol	1996	7.62	s60	NS	Nail	Unknown	5.60 × 16
s21	121	64 pistol	1990	7.62	s61	NS	Nail	Unknown	6.80 × 11
s22	121	92 rifle	2002	7.62	s62	NS	Nail	Unknown	6.80 × 11
s23	301	64 pistol	1980	7.62	s63	NS	Nail	Unknown	6.80 × 11
s24	301	64 pistol	1987	7.62	s64	Ω	Sporting rifle	Unknown	5.60
s25	311	64 pistol	1989	7.62	s65	Double Ring	Sporting rifle	Unknown	5.60
s26	311	64 pistol	1992	7.62	s66	△	Sporting rifle	Unknown	5.60
s27	311	64 pistol	1994	7.62	s67	△	Sporting rifle	Unknown	5.60
s28	311	64 pistol	2004	7.62	s68	YRD	Nail	Unknown	6.80 × 11
s29	611	56 rifle	1967	7.62	s69	YRD	Nail	Unknown	6.80 × 11
s30	671	53 pistol	2013	7.62	s70	YRD	Nail	Unknown	6.8 0× 11
s31	71	56 rifle	1956	7.62	s71	YRD	Nail	Unknown	6.80 × 18
s32	724	DAP92 pistol	2007	5.80	s72	YRD	Nail	Unknown	6.80 × 18
s33	791	9 mm pistol	2016	9.00	s73	YRD	Nail	Unknown	5.60 × 16
s34	791	56 pistol	1988	7.62	s74	YRD	Nail	Unknown	5.60 × 16
s35	791	SS109 rifle	2001	5.56	s75	YRD	Nail	Unknown	5.60 × 16
s36	791	DCV05 pistol	2008	5.80	s76	YRD	Nail	Unknown	6.30 × 16
s37	791	95 pistol	2016	5.80	s77	YRD	Nail	Unknown	6.30 × 16
s38	791	Pistol	2014	5.80	s78	YRD	Nail	Unknown	5.60 × 16
s39	791	DVP88A pistol	2017	5.80	s79	YRD	Nail	Unknown	5.60 × 16
s40	81	56 rifle	1964	7.62					

### GPC measurement

All SLPs were analyzed on the Waters 1515 GPC instrument (Waters Corp., Milford, MA, USA), consisting of a 2414 Refractive Index (RI) detector (Waters Corp.). In the experiment, the series connection of column of Waters Styragel HT3 (7.8 × 300 mm), Styragel HT4 (7.8 × 300 mm) and Styragel HT5 (7.8 × 300 mm) were used. The column and detedctor temperatures were both 35°C. The mobile phase was THF. The flow rate was ⁓1 mL/min. The collection time was 30 min and the sample volume for GPC measurement was 100 μL. Polystyrene standards (PS770-PS1350000) for GPC calibration were used. Each sample was measured with five replicates.

Inter- and intra-day precision evaluation was carried out using three different samples with sample number s1, s38, and s63. For intra-day precision, these three samples were measured five times in 1 day and the relative standard deviation (RSD,%) was calculated. The three samples were analyzed on five consecutive days and the data were used to evaluate the inter-day precision.

**Table 2:** Anlyzed mean values and relative standard deviation (RSD, %) of Mw, Mn and polydispersity index (PDI) of 79 smokeless powder samples.

Series number	Mw		Mn		PDI		Series number	Mw		Mn		PDI	
	Mean	RSD (%)	Mean	RSD (%)	Mean	RSD (%)		Mean	RSD (%)	Mean	RSD (%)	Mean	RSD (%)
s1	377 609	0.048	113 399	0.287	3.33	0.300	s41	342 815	0.058	126 978	0.131	2.70	0
s2	384 420	0.040	120 826	0.222	3.18	0.314	s42	351 666	0.046	133 901	0.191	2.63	0.380
s3	401 652	0.076	161 637	0.152	2.48	0.403	s43	313 484	0.057	93 209	0.245	3.36	0.298
s4	403 037	0.053	185 683	0.098	2.17	0	s44	340 849	0.086	139 437	0.083	2.44	0
s5	411 389	0.049	177 828	0.136	2.31	0	s45	342 363	0.036	117 984	0.265	2.90	0.345
s6	359 511	0.061	92 272	0.415	3.90	0.513	s46	281 623	0.069	106 987	0.206	2.63	0.380
s7	355 935	0.055	126 792	0.120	2.81	0.356	s47	330 161	0.061	116 413	0.230	2.84	0.352
s8	351 566	0.073	119 337	0.144	2.95	0.339	s48	301 918	0.076	110 671	0.223	2.73	0
s9	348 915	0.050	109 151	0.216	3.20	0.313	s49	258 148	0.072	106 540	0.191	2.42	0
s10	380 216	0.048	143 308	0.154	2.65	0.377	s50	307 260	0.099	110 372	0.207	2.78	0.360
s11	373 633	0.087	102 717	0.156	3.64	0.275	s51	294 956	0.103	96 073	0.227	3.07	0.326
s12	200 888	0.105	82 939	0.276	2.42	0.413	s52	322 763	0.085	165 853	0.150	1.95	0.513
s13	176 096	0.071	58 190	0.605	3.03	0.660	s53	284 187	0.068	88 221	0.319	3.22	0.311
s14	334 696	0.065	124 723	0.113	2.68	0.373	s54	283 987	0.091	82 817	0.333	3.43	0.292
s15	332 843	0.090	94 837	0.143	3.51	0.285	s55	326 248	0.065	117 499	0.139	2.78	0
s16	341 562	0.058	107 293	0.191	3.18	0.314	s56	290 207	0.071	87 382	0.208	3.32	0.301
s17	329 471	0.025	100 018	0.274	3.30	0.303	s57	256 346	0.087	76 481	0.235	3.35	0.299
s18	334 435	0.076	144 988	0.139	2.31	0.433	s58	282 871	0.053	101 802	0.200	2.78	0.360
s19	244 943	0.146	60 213	0.367	4.07	0.491	s59	260 301	0.095	79 791	0.150	3.26	0
s20	271 747	0.107	70 753	0.293	3.84	0.260	s60	294 477	0.041	103 959	0.181	2.83	0
s21	233 226	0.069	67 753	0.304	3.44	0.291	s61	235 659	0.076	71 735	0.236	3.29	0.304
s22	222 341	0.063	59 027	0.522	3.77	0.531	s62	262 356	0.048	85 378	0.166	3.07	0
s23	275 370	0.076	74 728	0.450	3.69	0.542	s63	248 470	0.043	79 579	0.388	3.12	0.321
s24	264 591	0.073	91 035	0.334	2.91	0.344	s64	279 183	0.111	93 356	0.310	2.99	0.334
s25	269 582	0.145	105 071	0.071	2.57	0	s65	255 071	0.097	65 005	0.328	3.92	0.255
s26	259 055	0.095	74 186	0.301	3.49	0.287	s66	237 862	0.110	71 234	0.458	3.34	0.599
s27	285 431	0.089	101 283	0.230	2.82	0.355	s67	264 637	0.079	71 254	0.431	3.71	0.539
s28	196 135	0.103	35 208	0.750	5.57	0.718	s68	275 158	0.100	83 773	0.267	3.28	0.305
s29	217 829	0.045	46 652	0.474	4.67	0.428	s69	301 821	0.068	100 090	0.244	3.01	0.332
s30	265 380	0.067	81 914	0.239	3.24	0.309	s70	292 491	0.071	104 005	0.312	2.81	0.356
s31	379 797	0.103	128 363	0.172	2.96	0.338	s71	255 273	0.091	69 449	0.304	3.67	0.272
s32	443 478	0.041	173 152	0.148	2.56	0.391	s72	286 906	0.105	75 951	0.371	3.78	0.265
s33	399 947	0.046	157 063	0.148	2.55	0.392	s73	287 967	0.090	88 019	0.309	3.27	0.306
s34	386 746	0.079	139 256	0.161	2.78	0	s74	282 806	0.092	83 101	0.183	3.40	0.294
s35	381 586	0.075	138 978	0.176	2.74	0.365	s75	278 265	0.100	95 752	0.255	2.91	0.344
s36	439 878	0.062	176 778	0.181	2.49	0	s76	304 709	0.087	107 305	0.213	2.84	0.352
s37	415 211	0.054	152 148	0.172	2.73	0	s77	305 580	0.105	105 267	0.190	2.90	0
s38	331 491	0.091	123 379	0.125	2.69	0.372	s78	236 087	0.125	70 598	0.384	3.34	0.299
s39	346 181	0.052	122 728	0.277	2.82	0.355	s79	309 538	0.058	101 229	0.169	3.06	0.327
s40	165 500	0.147	47 126	0.382	3.51	0.570							

Mw: weight-average molecular weight; Mn: number-average molecular weight; PDI: polydispersity index.

### LR calculation

LR calculation was applied in this work to give a quantitative evaluation of the analysis data. For comparison work, it was used to evaluate the strength of evidential data based on two contrasting hypotheses: (i) prosecution proposition (H_1_) proposed that the compared SLP samples came from the same source; (ii) defense proposition (H_2_) proposed that they had different sources. The general LR formula can be expressed by Eq 1:


(1) \begin{equation*} LR=\frac{f\left(E\ \right|{H}_1)}{f\left(E\ |\ {H}_2\right)} \end{equation*}

where, E is the evidence obtained from the Mw, Mn and PDI values. The definition indicates that values of LR > 1 support H_1_, while values of LR < 1 support H_2_. A value of LR = 1 does not provide support for either proposition. The magnitude (higher or lower) of the value of LR indicates increased support for the evidence (H_1_ or H_2_). The exact LR calculation model can refer to the paper published in 2023 by our group [13]. The LR model in this work was built based on the multivariate data of Mw, Mn and PDI of 79 SLP samples. LR calculation involving these three variables were calculated by programming with R software (https://www.r-project.org/).

The within-object distribution was assumed to be normal. The between-object distribution of the data was estimated by Quantile-Quantile (Q-Q) plot to check if they conformed to a normal distribution. Kernel density estimation (KDE) using Gaussian kernels was employed if between-object distribution could not be estimated by a normal distribution (for details see [14]).

## Results and discussion

### Mw, Mn, and PDI analysis of different SLP samples

**Figure 1:**
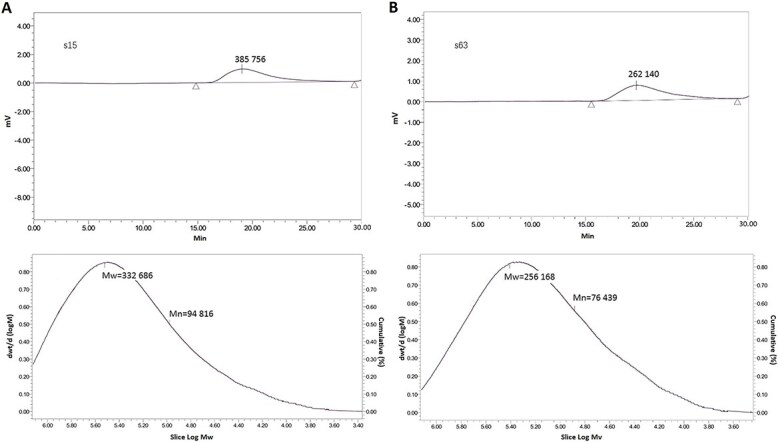
Chromatograms of samples s15 (A) and s63 (B). Mw: molecular weights; Mn: number-average molecular weights.

The inter-day precision for Mw, Mn, and PDI of sample s1, s38, and s63 was 0.052%, 0.295% and 0.368%, respectively; and the intra-day precision was 0.095%, 0.457%, and 0.579%, respectively, which demonstrated that GPC method had good reproducibility. Seventy-nine SLP samples were analyzed by GPC and the mean values of Mw, Mn and PDI were listed in Table 2. The GPC instrumentation can provide Mw and Mn at the same time, and PDI can be easily calculated. The PDI is used as a measure of the broadness of a molecular weight distribution of a polymer and is defined by $PDI=\frac{M_w}{M_n}$. The larger the PDI is, the broader the molecular weight disperses. In this study, significant scattering of Mw, Mn and PDI was observed: Mw ranged from 165 500 to 443 478, Mn ranged from 35 208 to 185 683, and PDI from 1.95 to 5.57. Figure 1 showed the chromatograms of samples s15 and s63, having different Mw, but close PDI (with a value ⁓3.3).

### LR calculation

**Figure 2:**
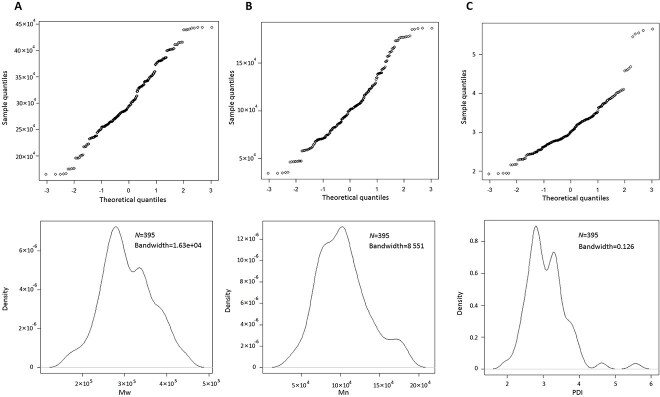
Q-Q plots and KDE curves for Mw (A), Mn (B), and PDI (C). Upper: Q-Q plots; Lower: KDE curves. Mw: weight-average molecular weight; Mn: number-average molecular weights; PDI: polydispersity index; KDE: kernel density estimation.

Q-Q plots and KDE methods were used to evaluate if the data conformed to a normal distribution. The Q-Q plots and KDE curves for Mw, Mn and PDI were shown in Figure 2. The probability distribution of the data may be assumed normal if the points lie close to or along the leading diagonal in Q-Q plot. According to the plots and curves of these three variables, they did not conform to a perfect normal distribution. So a KDE using a Gaussian kernel was carried out to estimate the probability density function for each variable. The LR values obtained combining Mw, Mn, and PDI variables on the within-source datasets were 5.72 *×* 10^2^ to 1.76 *×* 10^22^; the corresponding ranges on the between-source datasets were 0 to 0.443, with all the LR values <1. All the pairwised calculated LR values were listed in the Supplementary Table S1.

Forensic scientists are required to minimize evidential error rates since misleading LR values may lead fact finders to the wrong decisions in court. The following levels of support for H_1_ based on LR values have been reported [15]: 1 < LR ≤ 10 indicates limited support; 10 < LR ≤ 100 indicates moderate support; 100 < LR ≤ 1000 indicates moderately strong support; 1000 < LR ≤ 10 000 indicates strong support; and LR > 10 000 indicates very strong support. Making reference to the data in Supplementary Table S1, the LR values supported both hypotheses from moderate to very strong level.

**Figure 3:**
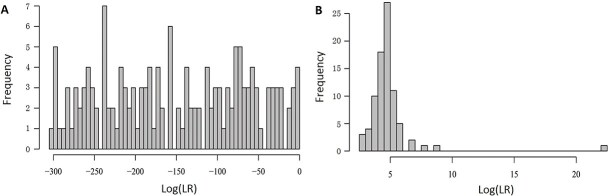
Graphs showing log_10_ (LR) distribution assuming KDE: true-H_1_ values (A) and true-H_2_ values (B). LR: likelihood ratio; KDE: kernel density estimation.

Positive and negative rate of misleading was calculated to evaluate the performance of the LR model. The positive misleading rate was evaluated by comparing the results obtained from two different projectile samples: there were ${c}_{79}^2=3\ 081$ pairs of comparisons; the desirable answer was LR < 1; each value of LR > 1 was considered a positive misleading answer. The negative misleading rate was estimated by forming two groups for a single SPL sample: Group 1 comprised two measurements (${n}_1$= 2); the remaining three formed Group 2 (${n}_2$= 3); the number of comparisons was 79; each value of LR < 1 was considered a negative misleading response. There were no positive or negative misleading results found in this work. The results were shown in Figure 3 and Supplementary Table S1, which indicated that Mw, Mn and PDI variabls and the LR model used were a good choice to make comparisons between SLP samples.

As a supplementary method to discriminate and individualize SLP samples, GPC made full use of the main component of NC no matter what kind of propellants they are, providing further information instead of focusing only on the additives in them. Mw, Mn and PDI information played a significant role, especially in cases where the additives of the compared SLP samples were the same. The LR evaluation method gave a quantitative evaluation to what degree that the evidence of support the two contrasting hypotheses H_1_ and H_2_. The method combining Mw, Mn and PDI measurement by GPC and LR evaluation has been used in real case sample comparisons with satisfying results.

## Conclusion

This work has demonstrated that Mw and PDI information was valuable to discriminate smokless powders from different sources, which was a helpful reinforcement to SLP commonplace analysis. Pairwise comparisons of 79 SLPs from different manufacturers and batches using LR computations gave no positive or negative misleading results. This study indicated that GPC, combined with LR calculation, can provide a promising way to discriminate smokeless powders.

## Supplementary Material

Supplementary_Table_S1_owaf005
